# Working toward sustainability: Transitioning HIV programs from a USA-based organization to a local partner in Zimbabwe

**DOI:** 10.1371/journal.pone.0276849

**Published:** 2022-11-10

**Authors:** Milan Vu, Marrianne Holec, Ruth Levine, Batsirai Makunike-Chikwinya, Jacob Mukamba, Scott Barnhart, Stefan Wiktor, Bryan Weiner, Caryl Feldacker

**Affiliations:** 1 Department of Health Services, School of Public Health, University of Washington, Seattle, Washington, United States of America; 2 International Training and Education Center for Health (I-TECH), Department of Global Health, University of Washington, Seattle, Washington, United States of America; 3 Zimbabwe Technical Assistance, Training & Education Center for Health (Zim-TTECH), Harare, Zimbabwe; 4 Department of Global Health, School of Public Health, University of Washington, Seattle, Washington, United States of America; 5 Department of Medicine, University of Washington, Seattle, Washington, United States of America; St John’s University, UNITED STATES

## Abstract

**Background:**

Despite the history of United States of America (USA)-based partners implementing global health programs in low- and middle-income countries (LMIC), future models for sustainable healthcare rely on local country ownership and leadership. Transition is the process of shifting programs towards country ownership, where local stakeholders plan, manage, and deliver health programs. Transition is not a singular event but a process which may include a phase where health programs are led and managed by local entities but still reliant on awards from international partners. This phase is scarcely described yet can impact long-term program sustainability if navigated poorly. This qualitative study examines the transition of Zimbabwe’s voluntary medical male circumcision and HIV care and treatment services from management by a USA-based organization, the International Training and Education Center for Health (I-TECH), to management under a new Zimbabwean organization, the Zimbabwe Technical Assistance, Training and Education Centre for Health (Zim-TTECH). The primary objective of this paper is to explore challenges, successes, and lessons learned during this transition to inform other non-governmental organizations.

**Methods:**

We conducted sixteen virtual, key informant interviews using purposeful sampling, identifying potential participants based on their role in the transition team (leadership, administrative, financial, or human resources) and willingness to consent to the study. We aimed for equal representation from USA-based, I-TECH headquarters staff and Zimbabwe-based, Zim-TTECH staff involved in the transition team. Data were analyzed in Atlas.Ti using deductive and inductive methods, followed by a thematic analysis guided by several frameworks for program transition and organizational change.

**Results:**

Findings suggest five themes to guide transition: 1) Develop a vision and empower leadership for change by delegating clear roles and supporting local ownership; 2) Plan and strategize for transition in a manner that accounts for historical context; 3) Communicate with and inform stakeholders to understand transition perceptions, understand barriers to transition, and enable open communications related to risks and benefits; 4) Engage and mobilize staff by constructing necessary infrastructure and providing technical assistance as needed; and 5) Define short-term and long-term success.

**Conclusion:**

Transition processes were challenged by the local country context, compressed transition timelines, and all-or-nothing measures of transition success. Facilitators included strong staff capacity and a synergistic partnership model between Zim-TTECH and I-TECH. Global funders and international organizations should support local LMIC partners in their pathway to independence by removing restrictions on funding awards, including transitioning ownership mid-stream, and positioning leadership of international awards for in-country entities.

## Introduction

Historically, United States of America (USA) -based partners have been primary funding award recipients of United States Government (USG) grants and stewards of global health programs in low- and middle-income (LMIC) countries, limiting the independence of local partners to lead health programs in their countries [1]. Future models for sustainable healthcare depend on local LMIC partners leading the way [2]. Country ownership is critical for efficient coordination of resources, optimized return on investment and long-term sustainability. Commitment to in-country ownership is increasingly recognized by major global health funders like the USA-based President’s Emergency Plan for AIDS Relief (PEPFAR) and the Global Fund to Fight AIDS, Tuberculosis, and Malaria [2, 3]. Likewise, a growing community of global health practitioners calls for international institutions and donors to decolonize global health and respond and respect the autonomy of LMIC countries to dictate the public health agenda and control funds. As the global AIDS response enters a new stage and is accompanied by shifts in funding priorities that award greater shares of resources directly to in-country LMIC partners, there is urgency to transition programs historically led by non-governmental organizations, for-profit organizations, and universities based in the Global North.

The concept of country ownership has evolved over time [4]. Per the Paris Declaration for Aid Effectiveness of 2005 and the Accra Agenda for Action of 2008, country ownership involves local partners leading the definition, design, and implementation of development priorities [5]. Collins and Beyrer [6] add financing of health programs as an additional component, while the Global Health Initiative summarizes country ownership as “the continuum of actions taken by political and institutional stakeholders in partner countries to plan, oversee, manage, deliver, and finance their health sector” [7].

Transition generally describes the process of shifting from external, donor-led programs toward domestic or locally-led program management and ownership [8]. Previous research from LMIC, including studies by Vogus [7] and Bennett [9], developed high-level frameworks for family planning and HIV programs to transition to country ownership. However, transitions that are disruptive or too rapid may negatively impact program and data quality, access to services, and risk the long-term sustainability of health programs [6, 9]. Intermediate transition steps, such as where local LMIC organizations lead and manage programs but continue to rely on USG-based funders, is little described in the literature or funder technical guidance and not well understood. This phase of transition, if navigated poorly, may pose similar threats to program sustainability. Organizational change management approaches, including that of Kotter [10], provide practical guidance for navigating these steps.

In this study, we focus on the process of transition from a USA-based, USG-funded entity to country ownership, specifically the transition of health programs or services to local LMIC organizations based in the country of program operation. We discuss the process of transferring PEPFAR-funded HIV programs in Zimbabwe from a USA-based organization, the International Training and Education Center for Health (I-TECH), to a local organization, the Zimbabwe Technical Assistance, Training and Education Centre for Health (Zim-TTECH). I-TECH is a global network housed in the University of Washington’s Department of Global Health that works with Ministries of Health and local partners to develop skilled healthcare workers and strong national health systems in LMIC countries. I- TECH promotes local ownership to sustain effective health systems and works with partner organizations to support the development of quality healthcare delivery systems. We assess factors that facilitated and impeded the I-TECH to Zim-TTECH transition by applying change management models and lessons from past global health program transitions. Our objectives are: 1) to inform transitions of other I-TECH programs; 2) to provide guidance for other non-governmental organizations (NGOs) that are entering similar processes; and 3) to advocate for USG identification of clear pathways for transitioning awards to local LMIC partners.

### Project background

The US Centers for Disease Control and Prevention (CDC) awarded two 5-year cooperative agreements for I-TECH to expand its training, mentorship, and implementation work in support of Zimbabwe’s HIV epidemic control efforts in 2013 [11]. At program inception, I-TECH partnered with a well-known research institution, the University of Zimbabwe Clinical Trials Research Centre (UZ-CTRC) to serve as the local hub for financial, administrative, and select logistical/programmatic needs rather than registering I-TECH as a local NGO in Zimbabwe. The scope housed under UZ-CTRC was referred to as, “I-TECH Zimbabwe”. On the intervention implementation side, I-TECH partnered with complementary local organizations to implement voluntary medical male circumcision (VMMC) and HIV prevention, treatment, care, and support services (C&T). Consistent with guidance in the CDC notice of funding opportunity for both awards, I-TECH formed two consortia to support implementation: ZAZIC (created from a combination of partner names) for VMMC in 2013 and ZimPAAC, the Zimbabwe Partnership to Accelerate AIDS Control, for C&T in 2018. For clarity, key acronyms and terms are presented in S1 Table.

**Fig 1:**
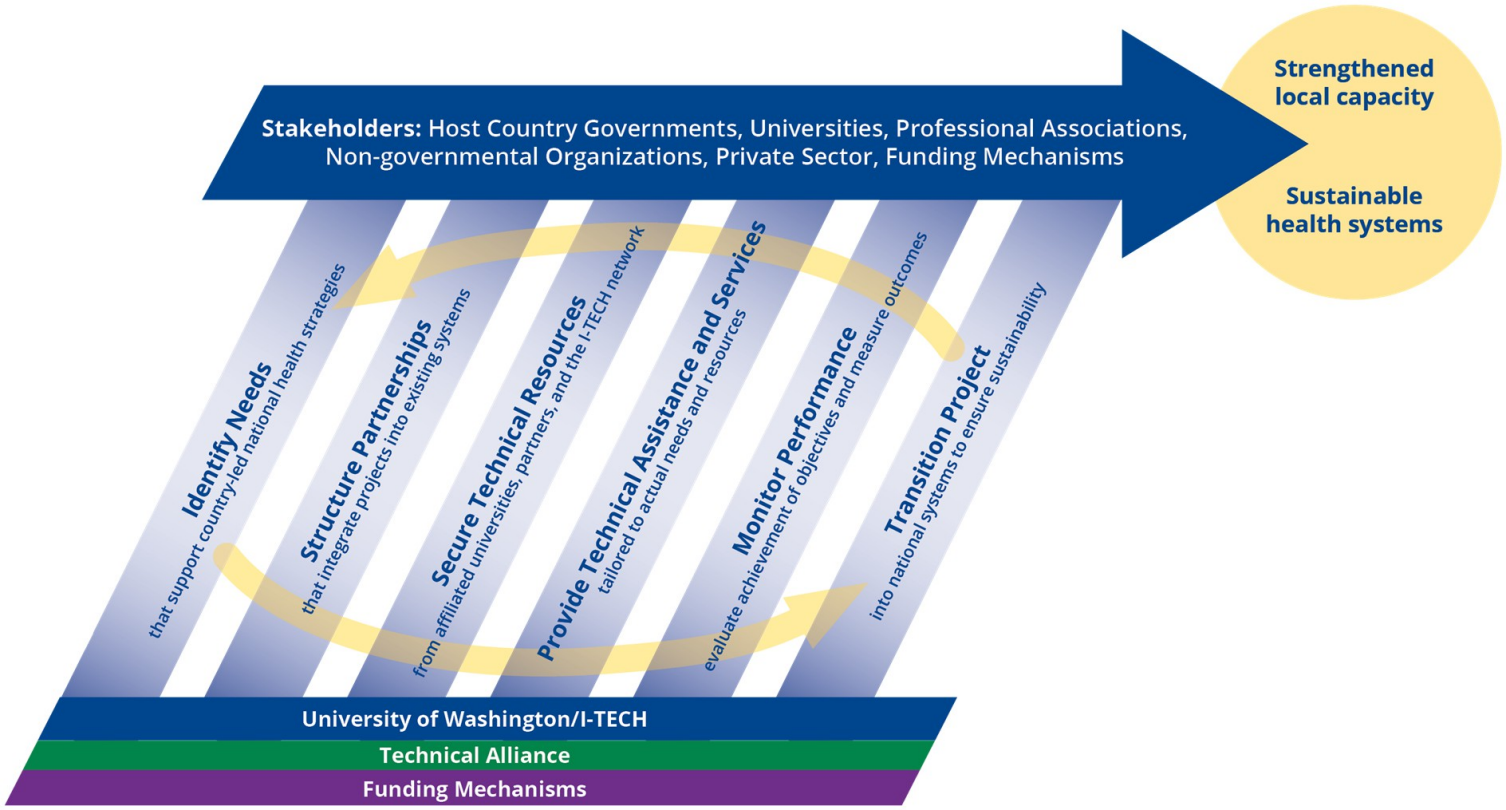
I-TECH’s partnership model.

Funding for VMMC and C&T was directed via a subaward to UZ-CTRC. In 2018, ZAZIC and ZimPAAC successfully competed for follow-on awards as local partners with I-TECH serving as Prime. *Prime* is defined as the primary recipient of USG funds that is responsible for programmatic and fiscal leadership and accountability [12]. Though I-TECH was Prime, UZ-CTRC served as the local lead on administrative functions of the VMMC and C&T awards and 90% of funding was directed in-country via the subawards. This arrangement was reflective of I- TECH’s partnership model (Fig 1) which emphasizes local organization capacity strengthening as a primary objective of global health practice.

In July 2018, as part of a strategy to shift towards program sustainability, PEPFAR set targets to direct 40 percent of PEPFAR funding to local organizations within 18 months and 70 percent of funding in the subsequent 30 months [13, 14]. Based on PEPFAR’s new guidelines, CDC realigned the criteria for a *local* organization in Zimbabwe, including a stipulation that a local entity must be legally registered as an independent Zimbabwean organization [15, 16]. This new definition disqualified I-TECH Zimbabwe’s consortia from being considered local since it was not registered independently. With widespread recognition that future PEPFAR funding would give strong preference to local organizations and seeing the alignment with I-TECH’s own partnership model, I-TECH, I-TECH Zimbabwe and UZ-CTRC embarked on a process to determine the best path forward. Three paths were considered: 1) transitioning Prime to UZ-CTRC, 2) looking for another local organization to serve as Prime, or 3) establishing a new, separate financially and administratively independent local entity that could eventually become Prime and fit the CDC definition of *local*.

The team ultimately chose pathway three: to establish a new, separate, local entity to respond to the demands and urgencies of program implementation. While reactive planning to the PEPFAR strategy shift may entice USA-based organizations to establish locally-based entities while maintaining external oversight, I-TECH sought to help ensure that the new entity was truly an independent local organization. Evidence of this local independence included: local legal registration; establishing its own board without individuals from I-TECH headquarters (HQ); making independent and autonomous staffing decisions; independently deciding on whether and which funding opportunities to respond; and developing other funder/partner relationships outside of I-TECH. Staff operating under the previous organization (employed by UZ-CTRC and operating under the program “I-TECH Zimbabwe”) were fully transitioned to the new organization (Zim-TTECH), including transitioning the Zimbabwean Country Director for I-TECH Zimbabwe to the new role of Executive Director, Zim-TTECH. The intention of this new entity was to succeed as a separate organization and diversify, regardless of the future trajectory of I-TECH or the Seattle-based supporting teams.

By fall of 2018, I-TECH initiated the process of focused capacity strengthening of Zim-TTECH as an independent, autonomous organization with the eventual goal of transitioning management of the VMMC and C&T awards to them as a local organization. Between September 2018 and June 2020, the transition team (composed of I-TECH HQ staff and I-TECH Zimbabwe, subsequently Zim-TTECH, staff) developed the needed human resources (HR), operations, and fiscal infrastructure for Zim-TTECH to function as an independent NGO to increase Zim-TTECH’s readiness to compete as Prime. The transition team jointly established new procedures for sharing award management functions between Zim-TTECH as Prime and I-TECH HQ (S2 Table).

## Methods

### Study design and participants

Due to COVID-19 related constraints on international travel and in-person interaction, key informant interviews (KII) were conducted via Zoom video conferencing for this qualitative study. We selected KII participants using purposeful sampling [17], including staff based on their role in the transition team (leadership, administrative, financial, or human resources), willingness to consent to the study, and targeted similar numbers of representatives of USA-based, I-TECH HQ staff and Zim-TTECH staff, all of whom are Zimbabwean. No additional demographic factors excluded participants from engaging in the study. We followed the Consolidated Criteria for Reporting Qualitative Research (COREQ) to describe our methodology [18].

### Data collection

An interview guide was developed in collaboration with I-TECH HQ and Zim-TTECH transition staff, using Bullock and Batten’s [19] change management framework as a basis to ensure interview prompts touched on key change processes. The interview guide covered the following topics: the participant’s role in I-TECH HQ or Zim-TTECH, the participant’s views on the decision to transition, transition planning, transition implementation, technical support for transition, advice for other organizations considering transition, ethics of transition, and defining transition success. A senior researcher (study principal investigator) and junior researcher conducted interviews in English over Zoom video conferencing between June-July 2020. Written consent to participate in the study was obtained prior to the video conference and confirmed verbally at the onset of the video conference.

Participants were provided with a brief introduction describing the purpose of the study at the outset of each interview. Participant demographics of interest included organization affiliation (I- TECH HQ vs. Zim-TTECH), organization role, and years of experience with the program. Interviews were semi-structured and questions were tailored based on the participant’s organization affiliation and role to enrich responses on topics that aligned with the participant’s knowledge areas. All KIIs were conducted in English, recorded, and ranged from 30 to 75 minutes in duration. Reflexivity statements were noted prior to each interview to record the researchers’ experiences, assumptions, and beliefs that could influence the research process. KII transcripts were auto-generated using Zoom’s built-in transcription function. Due to the presence of gaps or garbled text in the auto-generated transcripts related to periods of poor audio quality, connectivity glitches and/or background noise, each transcript was verified for accuracy against the audio recording by the interviewing investigator and two USA-based I-TECH HQ staff. Early themes were presented to study participants to validate, inform, and explore the findings.

### Data analysis

Two researchers coded the interview transcripts in Atlas.ti 8.0 using a hybrid deductive and inductive approach [20]. Several transition frameworks (Table 1) were considered to help structure the transition process, inform the interview guide, and suggest a priori codes.

**Table 1:** Comparison of transition-relevant frameworks.

Vogus’ key steps in transitioning to country ownership [7]	Bennett’s central themes to support orderly program transition [9]	Kotter’s sequential process for leading change [10]
Develop a roadmap	Develop a vision, and mobilize commitment and leadership for change	Establish a sense of urgency
		Create a guiding coalition
	Craft an implementation plan or strategy	Develop a vision and strategy
Invest in stakeholder participation	Communicate and inform stakeholders	Communicate the change vision
Communicate the plan through high-level diplomacy		
Provide technical assistance throughout the process	Engage staff	Empower broad-based action
Support midterm evaluations	Generate short term wins	Generate short term wins
Provide long-term monitoring and evaluation (M&E) support		Consolidate gains and produce more change
		Anchor new approaches in the culture

### Ethics statement

This human subjects study, “Transitioning from international to local NGO: lessons learned from the Zimbabwe I-TECH to Zim-TTECH process (IRB ID: STUDY00010422, PI: Feldacker) was granted exempt status by the University of Washington institutional review board due to its focus on internal program processes and improvements. As part of written consent, all participants were informed that their participation was voluntary, confidential, and would have no impact on their employment with I-TECH now or in the future. No compensation was provided for participation. Video and audio recordings were deleted once data were transcribed and verified. Names were excluded from the final transcripts used for analysis.

## Results

### Develop a vision and empower leadership for change

#### Develop a vision

Though many respondents reported that donor mandates to prioritize and direct funds to local organizations prompted the transition, local ownership and management of programs was long nurtured as a core value of I-TECH and the Principal Investigators (PIs) of both cooperative agreements. One I-TECH HQ member describes:

“This term local ownership, or who is the principal recipient and who is the driving force of a program, is an issue. Within I-TECH, transition to local ownership has been a principle…a high-level philosophy. It is something we all think is important and need to aspire to and move towards…We’re not supposed to be keeping ourselves going.”

Respondents noted that both the VMMC and C&T programs practiced this intention by directing program administration through UZ-CTRC as a local sub-award partner, rather than through the standard I-TECH mechanism–an in-country I-TECH office–at the initiation of the first cooperative agreements in 2013. As a result, the majority of funds were directed to existing Zimbabwean organizations, effectively minimizing I-TECH’s presence in terms of administrative space, infrastructure, and non-local personnel.

### Empower leadership for change

In the transition, roles were intentionally distributed: I-TECH HQ as the project coordinators, ideologic champions, and support staff and I-TECH Zimbabwe staff held decision-making authority and led implementation of the transition on the ground. Doing so helped Zim-TTECH employees accept the transition, as one Zimbabwean respondent describes:

“What I liked about it is that they [I-TECH HQ] did not take ownership of it…Unlike if they had owned the process and then throw it back to us, there would have been some resistance…In terms of policy making, there’s more of a guiding approach to everything which I thought was a very good thing because then we also took ownership of the process.”

Zim-TTECH respondents noted the need for having I-TECH HQ involved during the transition, particularly as in-country staff balanced the dual burden of day-to-day program operations and transition activities. This function is explained by one participant as:

“I think the problem is that we are so much of an implementer that we don’t have in our system that role of someone who steps back and says, “There are other things we need to be doing. And [name], you’re doing this. Let’s get it done.”

### Plan and strategize for transition

#### Plan for transition

After supportive discussions with CDC, and consistent with the prioritization of country ownership by PEPFAR, transition planning was kicked off by a week-long meeting that included representatives from the I-TECH executive team, I-TECH HQ staff, and Zim-TTECH’s Deputy Director. This meeting yielded an opportunity for the transition team to assess the situation, identify critical transition infrastructure, specify timelines, delegate roles, and generate a tangible transition plan referred to as the ARCHI chart (S2 Table). Staff also deliberated the new organizational relationship between Zim-TTECH and I-TECH HQ. The planning meeting provided motivational value as an attendee reflects:

“I could have just been swallowed by the fear, by the lack of faith, and the doubt that was around me locally…That week I spent in Seattle was really a boost as well. We sat down with the team and people were just ready to do it.”

Early, in depth planning was also key to successfully navigating the transfer of employment contracts and other documentation from UZ-CTRC to Zim-TTECH. One respondent specializing in HR noted:

“You need to have roadmaps on what exactly you intend to achieve within what time because, especially from an HR perspective or from a contracts dimension, it’s a highly legal area. If you muddle around with it, you drag the company into a huge financial liability.”

### Supportive context in Zimbabwe

Numerous respondents remarked on the capabilities of the Zimbabwean team, particularly the high level of technical expertise and full understanding of the complex political and regulatory environments of the VMMC and C&T programs. Several respondents noted that an in-country leader was vital to champion the transition, interface with I-TECH partners in Zimbabwe, and connect with stakeholders when transition-related issues arose. In addition, a seven-year history of collaboration between the Zimbabwe team and I-TECH HQ developed strong relationships and a sense of mutual trust noted by many of the respondents. This helped to facilitate ease in communication, staff mobilization, and jumpstarted several transition processes.

“The good thing is over the years, I-TECH and I-TECH Zimbabwe were growing. We were kind of already setting a transition in motion because we are now recruiting almost parallel systems with what existed in the parent administration organization, UZ- CTRC…So we were drifting towards our own independence. I think that’s what made it easier to transition, not by design but because of the nature of increasing amount of work and amount of funding.”

### Communication

#### Understanding transition perceptions

Reactions to transition spanned from supportive to hesitant, some “excited about the change because it meant more independence and freedom to run the programs exactly as they felt were necessary” while others “felt the current model was effective, advantageous and had worked well and resulted in program success.” Among Zimbabwe staff, apprehensions arose due to some associating transition with downsizing, reduced funding, and potential job loss. While not entirely unwarranted, one individual noted, “When they see staff going, they may attribute it to transition by itself. But it’s a direct result of a cut of funding, which could have occurred even if we had stayed as an international organization.” I-TECH HQ staff also had concerns about roles declining or going away, and around employee morale if “by doing this [transition]… am I basically working my way out of a job?”

#### Open communication on transition risks and benefits

At the employee-level, several Zimbabwe staff felt more confident working under I-TECH as an established, international organization with a track history of mobilizing resources. Working for Zim-TTECH brought uncertainty around job stability, stability of remuneration currency, poor quality recruiting and staffing standards, and susceptibility to Zimbabwe’s political and economic environment. At the institutional level, a couple I-TECH HQ respondents noted the potential loss of salaries for HQ staff on both VMMC and C&T awards. I-TECH HQ respondents also noted the risk of losing locations for additional program implementation, research, and layering on of work in Zimbabwe, fears about the loss of relationships with in-country partners, and loss of control over the operations and success of the programs. HQ respondents also mentioned some lingering reservation about relinquishing oversight of Zimbabwe employee contract compliance, sponsor reporting, budget reporting, efficient use of funds, and procurement to the new Zim-TTECH team that, while highly experienced, was still unproven in the capacity of Prime.

Yet, by one respondent’s assessment, the benefits to establishing a new, locally based organization outweighed the risks associated because “Zim-TTECH is able to have two things: greater access to local funding opportunities and greater autonomy from the University [of Washington] system.” Multiple respondents perceived local institutions to have more flexibility, as regulations on funds and resources are determined by the type of organization created (ex. a trust or private voluntary organization) and country laws rather than the regulations governing USA-based partner organizations like I-TECH and their affiliated academic institutions. Fewer restrictions may yield greater responsiveness to dynamic changes in the local environment.

Across respondents, transparent communication about risks and benefits up and down the chain of command and creating open feedback channels was key to quelling fears, meeting milestones and timelines, explaining new processes, and overcoming difficulties encountered in the transition process. One respondent in a management role explains, “that hearts and minds component, and making sure that people are really on board is the difference between teetering on success and failure.”

#### Communication with stakeholders

Though some saw the transition as a natural consequence of shifts in the funding environment towards local organizations, leadership on the transition team intended transition as a “deliberate approach and one that is clear on the direction with all the stakeholders, donors, staff, and boards.” Zim-TTECH leaders actively engaged stakeholders from the Zimbabwe Ministry of Health and Child Care (MoHCC), including the Permanent Secretary at the national level and Provincial Medical Directors at the subnational level, ZAZIC and ZimPAAC consortium partners, and donors. Established trust between Zim-TTECH and the Zimbabwe MoHCC reassured partners that the transition would advance the interests of people living with HIV, as opposed to self-interest. Several respondents remarked that they communicated about the transition as simply a change in name, and not in scope of work:

“For the program directors out in the field, things haven’t changed that much. I mean their identity has changed from UZ-CTRC to Zim-TTECH…The most significant changes were for the central staff to manage the finances, scan for opportunities, and create collaborations to allow discovery of funding opportunities. Stakeholder confidence in the transition came in once this was understood, and with reminders from Zim-TTECH that we were still the same organization.”

Though Zim-TTECH staff led communications to Zimbabwean stakeholders, one respondent felt that supportive messages coming from international partners would have aided their efforts. “For the Ministry partners to hear from CDC and from I-TECH HQ, ‘Yes, of course, we want to transition. They’re ready’…would ease the process…So that they also understand it’s what is required by the donor.” In retrospect, several respondents from both I-TECH HQ and Zim- TTECH also highlighted the need for Zim-TTECH to take a more active role when it came to communications with USA-based CDC representatives and for Zim-TTECH staff to proactively, and confidently, assume responsibility for direct interactions with the funder.

### Engage and mobilize staff

#### Construct necessary systems and infrastructure

The process of establishing Zim-TTECH as an independent, local organization required legally registering with the government of Zimbabwe, creating an Advisory Board as required of a registered trust, and developing administrative systems. Many respondents reinforced the significance of finding a local legal team to guide Zim-TTECH through these processes. One Zimbabwean respondent illustrates the need as such:

“Get a lawyer, you can work with…a lawyer who’s open enough to tell you that, ‘Look, the system takes forever. But we’re nearly there and I’m holding your hand’…Don’t take this person on board because [they are] from our circles, we’ve heard they’re linked to such and such political party; ordinarily that causes problems in this country. You want a lawyer who’s advisory and not a lawyer who just bills the hours.”

Determining how to register Zim-TTECH as an organization was described by many as complex and time-consuming. Despite preference for registering as a private voluntary organization, the timeframe mandated by the funder for this transition–2 years–limited the independence pathway to that of a Trust. Numerous respondents commented on needing a longer timeline for the transition process to allow a more gradual migration to becoming an independent organization.

Additionally, ensuring strong fiscal systems was key to establishing an organization. However, in the context of Zimbabwe’s volatile banking infrastructure, the transition team was consistently challenged to design, implement, and ensure robust fiscal systems for Zim-TTECH to minimize exposure to potential fraud, and abuse. Balancing the pressure between staff capacity limitations and required, multi-level assurances for sound fiscal systems is an ongoing challenge.

#### Provide technical assistance

Throughout the transition, I-TECH HQ offered high-level technical assistance that drew on past transition experiences from Haiti, India, and other partners within the I-TECH network including HQ staff. Staff training and mentoring for transition addressed key operational responsibilities of an award Prime, including applying for awards, navigating USG funding requirements, and developing expertise, staffing, and systems to fulfill complex financial reporting requirements. One Zimbabwean respondent noted:

“The actual applying, filling in those CDC forms–none of us have ever done that before. And those forms came out to 800 pages for the full submission. So that was a culture shock for us…because there is so much language around American forms or American processes that we would not ordinarily have known.”

Several respondents remarked on the value of in-person interaction to transfer experience, particularly for complex tasks such as compliance checks, financial reporting, budget forecasting, transaction management, and fund management. In-person interactions increased rapport and clarity of instructions, as some mechanics did not translate well over email or video conferencing. One respondent adds, “usually the impression that you get from virtual support is not necessarily what you find on the ground. Most of it is not deliberate but may mostly do with perception. What we are calling X, is it really X or to someone who can see it is it Y?”

Some respondents felt implementation could have moved faster if I-TECH staff were able to provide on-the-ground consultation to their counterparts in Zimbabwe, partly because “the ability to be there in-person demands some attention, so people shift their time to it.” Though virtual contact could not replace physical presence, virtual channels could supplement the support typically given during on-site visits and provide a mechanism for long-term mentorship or one-on-one development.

### Define short term and long-term success

Overwhelmingly, the primary marker of transition success, both short and long term, was identified as Zim-TTECH winning and receiving funding. However, despite well-known and respected Zim-TTECH capacity, many expressed fears of funding shortfalls. One Zimbabwean respondent noted common misperceptions some international funders may have about local organizations, and the idea that “local institutions sometimes fall victim to resources, deficiencies in corporate governance, corruption and even theft…If we are painted with the same brush, people may assume every local institution has no capacity to operate independently.” Overcoming these doubts about sustained, independent funding is a significant hurdle in creating confidence for a successful Zim-TTECH future.

Beyond this immediate funding target, multiple respondents spoke to a new vision for the organization characterized by name recognition and high performance. One Zimbabwean respondent shared, “My vision is an established organization that is known for what it does…Zim-TTECH will house ZAZIC, it will house ZimPAAC, it will be the health powerhouse…It’s going to be a self- sustaining organization that has the capacity to do research.” Another said, “through our policies, we are expecting the best of behavior, the best of performance, the best impact on the ground.”

## Discussion

In this paper, we explore factors that challenged and facilitated the transition process for the Zimbabwe VMMC and C&T programs from USA-based management by I-TECH HQ to local management under a new organization, Zim-TTECH. This step, where local organizations manage health programs funded by USA-based or other external funders, is a significant component of larger transitions to country ownership, excluding taking over the responsibilities as the Prime, that should be navigated with care. The findings from this study highlight several considerations for organizations moving towards country ownership, namely: account for the country context with respect to economy, governance, and organizational culture; assess capacity for transition before proceeding; provide ample time for transition processes; and bring equitable balance to the partnership between local organizations and USA-based partners. This study suggests that transition timelines and indicators of transition success were challenges while existing local capacity and fundamental support for local organizations aided the transition process. In addition to these key outcomes, we reflect upon the utility of applying existing transition frameworks to transition in this short- term context. We apply a change management lens to suggest additional steps left in the transition and call on funders to evaluate their role in facilitating transition processes.

First, funders and implementing partners considering transitioning should reflect on the minimum time needed to achieve the goals of each transition phase. The need for an extended and well-sequenced timeline is a recognized facilitator of success among organizations undergoing transition [8, 21, 22]. For Zim-TTECH, the major milestones for transition are: 1) Zim-TTECH is established as a trust; 2) I-TECH HQ, as Prime, sub-awards to Zim-TTECH; 3) Zim-TTECH is established as a private voluntary organization; 4) Zim-TTECH is Prime and sub-awards to I-TECH HQ for technical assistance. Each phase of the Zim-TTECH transition is highly complex, involving numerous prerequisites to achieve, and was largely supported by pre-existing organizational capacity, staff capacity, technical proficiency, and stakeholder relationships built before the timeframe for this transition was set. Luckily, much of the transition work happened before COVID-19 restrictions on travel and in-person meetings; however, the value of in-person support for this sensitive and complex work should not be discounted nor underestimated. If trying to achieve similar outcomes via virtual communication, only, the timeline would likely extend. Funders and coordinating partners should therefore not underestimate the time needed to prepare organizations for successful transitions or overlook the risk that accelerated timelines poses to both the transition process and program operations.

Another challenge is defining the markers of successful transition. Although program monitoring and evaluation (M&E) for HIV-related programs is commonplace, M&E of the transition process is poorly developed. A few studies offer suggestions for measuring transition success [21, 23, 24], including indicators to note the specific transfer of financial responsibilities from donor to recipient and progress towards financial stability [25]. However, these indicators do not include the primary marker of success according to Zim-TTECH: Zim-TTECH winning USG grants as Prime. In Zim-TTECH’s case, this milestone was presented as a dichotomy: they win (Zim-TTECH lives) or they do not (Zim-TTECH ceases to exist). Loss of funding, risks related to managing multiple funding streams, and challenges with diversifying funding are all threats to Zim-TTECH’s success and warrant some degree of mitigation. One-on-one mentorship and incremental steps to build grant-writing functions should be pursued to support Zim-TTECH’s capacity for achieving successful future funding.

High capacity among the Zim-TTECH team aided transition processes both technically and interpersonally. Though capacity is frequently cited as critical to overall transition processes, perhaps overlooked is its role in minimizing consequences of transition activity on HIV service delivery [9]. In addition to leading the transition process itself, strong Zim-TTECH leadership capacity meaningfully improved stakeholder perceptions of the transition, including signing an independent Memorandum of Understanding between Zim-TTECH and the MoHCC to ensure services continuity and preparing Zim-TTECH leaders (Directors, managers, coordinators, etc.) for the ways their roles would change as executive leaders of an independent NGO. Local organizations and affiliated partners considering transition would benefit from applying organizational assessment tools or contextual analyses to highlight areas of strength and/or weakness with respect to capacity development before moving forward [26]. Staff could then benefit from additional training where gaps or weaknesses have been identified, including in areas of leadership and management, to ensure sufficient capacity to move forward.

Another facilitator of the Zim-TTECH transition stems from I-TECH’s support for Zimbabwean entities through sub-awards to local organizations, as opposed to establishing an I-TECH office to manage VMMC and C&T programs. Partnering, and prioritizing local partner leadership from inception, put into practice I-TECH’s stated values for a locally-led response and directed funds to in-country partners early on, setting up the potential for subsequent transition. These initial steps facilitated the development of a synergistic partnership modeled between Zim-TTECH staff and I-TECH HQ that aided transition processes. Considering the cumulative workload transition places on local partners, plus the absence of dedicated funds or new staff to support the process, such partnerships and delegated roles have practical advantages for helping alleviate the load on local implementing partners. However, USA-based partners in similar roles as I-TECH HQ must be mindful to not encroach upon the jurisdiction or autonomy local actors have in the transition process. Intentional, synergistic partnerships may also help set the stage for future peer-to-peer relationships and offer the opportunity for both organizations to bring their best practices to partnership during and after transition.

In comparing key transition steps from this study to those described in Bennett’s [9] and Vogus’ [7] frameworks for transition, similar key themes are identified but with varying degrees of importance. Communicating with and engaging stakeholders had lesser significance for Zim-TTECH relative to other steps, given the minimal effect this transition would have on partners in the field and their communities. In comparison, transitions that shift large portfolios of donor-supported programs to country governments require far more in terms of diplomacy, stakeholder engagement, and communication due to differences in scale, level of investment, and negotiation needed to change over to government management and financing at the national level [9]. Separately, while the sequence of transition steps is not explicitly addressed by this study, change management literature asserts a strong case for a progressive transition process. The transition process concludes not with short-term wins but after gains are anchored in the organization’s independent culture [10]. In later phases of a change process, resistors to change, old norms, and previous linkages can threaten the sustainability of the change process. How this applies to I-TECH HQ and Zim-TTECH warrants consideration as Zim-TTECH moves forward and seeks to establish its independence, new processes, relationships, and vision. For future transitions, blending approaches from international development with those from change management would help organizations holistically plan, execute, and evaluate the complete life cycle of the transition process.

## Limitations

There are several limitations that affect the application of this study towards future or similar transition efforts. First, this transition takes place in Zimbabwe. The larger country environment places unique risks for the transition teams in both the immediate and long-term and may be quite dissimilar to other contexts and countries. Moreover, the respondents selected for participation in the study were restricted to I-TECH HQ or Zim-TTECH staff, which may not be representative of all stakeholders involved the transition process including UZ-CTRC staff who were not transferred to Zim-TTECH, funders, and the MoHCC. Similarly, as experts on the process, members of the transition team informed the KII guide, a benefit and a bias that influences the study findings. Also, the transition from I-TECH to Zim-TTECH had the full support of the PIs who set the vision for local ownership at the beginning of both cooperative agreements; this enabling environment may not be present in other program contexts. Furthermore, cultural bias also places this conversation about transition through a Western lens, and filters assessments of leadership and management practices for transition through frameworks developed by authors affiliated with Western countries. Additionally, the scale and scope of this study cannot reflect the full intricacies of financial, legal, and administrative management and regulation in the transition process. For example, the study did not take into consideration the issue of limits on USG-funding for indirects (overhead) to foreign institutions, which is currently capped at 10%. This arbitrary limit is a barrier for local organizations to ensure robust administrative operations.

Importantly, despite I-TECH’s assessment that Zim-TTECH was well positioned to assume responsibilities as Prime and extensive discussions with CDC as the funder to transfer Prime of VMMC to Zim-TTECH, the CDC Office of Grants Services (OGS) could not identify a feasible path to transition the Prime within the terms and remaining two years of the grant. The inability of the funder to identity a path for formal transition of the Prime represents a critical shortcoming that underscores the complexities of transitioning responsibilities across HR, operations, fiscal, and technical domains. The inability of CDC to find a path for Prime transfer also limited the extent to which transition could be fully tested and evaluated. Therefore, it is unknown whether the steps identified for transition are both necessary and sufficient. Despite these limitations, the authors believe that the contribution to the transition literature and the benefit of these shared experiences outweigh the limitations of these findings for others considering this complex, but critical, process.

## Conclusions

While the immediate recommendations stemming from this paper target local organizations and their international or USA-based partners, we also must recognize the responsibility funders and USG decision-makers have to enable smooth transition processes. Particularly as more funding shifts to local partners, donors must consider if the existing funding mechanisms, procedures, and requirements previously navigated by large, USA-based NGOs can be successfully completed by local organizations competing as Prime for the first time. Donors should also consider whether grant-making processes are accessible, equitable, and do not impose unnecessary barriers to local applicants. Donor policies should support the ample, indirect cost recovery needed to ensure local investments in infrastructure and fiscal resiliency (e.g. reserves) to promote sustainability and not dependency. In setting up future health programs, funders and global health leaders should also consider who is best served by the current organizational business models, policies, and practices. To truly center local country organizations and leaders over external preferences and power, the global community must work internally to break down these established systems for global health programming implementation to better enable and support country-led health program, policy, and priority setting. Only through success in these areas will global health move towards the promise of decolonizing global health.

## Supporting information

S1 Table Acronyms and terminology. (DOCX)

S2 Table ARCHI transition task tracker. Transition plan t for Zim-TTECH transition.
(DOCX)
