# Supplementary material for: Working toward sustainability: Transitioning HIV programs from a USA-based organization to a local partner in Zimbabwe
Source: PLoS One. 2022 Nov 10;17(11):e0276849. doi: 10.1371/journal.pone.0276849 (PMC9648773; doi:10.1371/journal.pone.0276849)
Supplement: S1 Table — (DOCX) [file pone.0276849.s001.docx]

# S1 Table: ARCHI Transition Task Tracker

| **Type** | **Activity/Task** | **Priority (1,2,3)** | **Percentage Complete** |
| --- | --- | --- | --- |
|  | *Review of ALL administrative policies & SOPs for Zim-TTECH* | 1 |  |
| Legal | Ex. engage an attorney; develop legal counsel policy |  |  |
| Trust Registration | Ex. create trust name; determine founders, trustees, board members, trust objectives |  |  |
| Private Voluntary Organization Registration & Board Development | Ex. determine board structure; develop bylaws; develop board capacity | 1 |  |
| Organizational Development | Ex. develop vision, mission, organizational structure, management framework, strategic plan, and business plan for new entity; develop and issue Zim-TTECH subaward. | 3 |  |
| Awards/Grants Management | Ex. develop policies, procedures, and SOPs for award management; identify authorizing official for new entity; register with Grants.gov, Sam.gov, DUNS | 3 |  |
| Pre-Award | Ex. develop decision making process for pursuing opportunities, sub-award selection and cost reasonableness review, standard allocating method for costs across multiple awards and standard documented rates. | 3 |  |
| Post Award | Ex. develop process for award review, negotiation and acceptance; identify staff and process for liaising with sponsor administrative arm and submission of prior approval requests, tracking and submission of programmatic activity reports and annual continuations. | 1 |  |
| Subawards | Ex. develop subawards policy and SOPs, contract templates and flow down provisions for funding sources to align with local laws and U.S. Uniform Guidance, management, monitoring & visit strategy and calendar. | 3 |  |
| MOUs | Ex. develop MOU templates; identify persons authorized to sign MOUs on organization's behalf; identify situations where additional legal review of MOUs is required. | 1 |  |
| Budget Management | Ex. finalize budget management policies and SOPs; implement budget management systems/templates/processes; determine standard reports and reporting dimensions for budget management. | 1 |  |
| Finance Organizational Model | Ex. determine finance team structure; determine fiscal year reporting period; contract with external audit firm; register with tax authorities; customize payroll software to entity needs. |  |  |
| Funding | Ex. establish bank accounts with appropriate signatories and authorizations; set up petty cash account; identify mechanism for advance/working funds for prime and subs. | 1 |  |
| Finance Operations | Ex. establish internal controls; set up financial record accounting systems; implement finance tasks such as posting transactions, paying vendors, invoicing, accounts review/reconciliation, depositing funds, closing fiscal periods, generating monthly and annual financial reports. | 1 |  |
| Records Review & Retention | Ex. purchase fireproof safe for all official documents; purchase separate locking safe for petty cash; develop records retention policy. |  |  |
| HR | Ex. establish HR policies, procedures, and SOPs; establish salary scale, compensation philosophy, and procedure for reviewing and establishing benefit rates; create and implement recruitment plan to hire staff; train staff on Zim-TTECH HR policies/SOPs. | 1 |  |
| Procurement | Ex. develop procurement policies and procedures; define roles and responsibilities ensuring separation of duties; establish procurement committee (bids above $20k). |  |  |
| Travel | Ex. develop travel policies, procedures and SOPs and all forms; establish local per diem rates |  |  |
| Vehicles | Ex. develop vehicle policies, processes, SOPs and forms; develop fleet management policies, procedures and system; purchase/transfer vehicle insurance. |  |  |
| Safety & Security | Ex. develop Security, Safety & Emergency Policies and SOPs; transition vendor agreement with security firm to new entity | 1 |  |
| Facilities | Ex. transition lease, utilities, phone service agreements, internet service, and guard service to new entity |  |  |
| Equipment  (> $5,000) | Ex. verify current equipment and supply assets; determine country laws or partner expectations; obtain funder prior approval for equipment with a current market value of $5,000 or more that is being sold/donated/turned over. |  |  |
| Insurance | Ex. survey market for insurance providers; identify & purchase mandatory and optional insurance, including employee medical and life, professional liability, facility and auto. |  |  |
| IT | Ex. develop IT policies, procedures, and SOPs; develop Knowledge Management, Back Up, Website Management, Change Management and User Account SOP's. | 1 |  |
| Communications & Branding | Ex. develop communication policies and SOPs; identify communications point person; develop logo; assure new name and logo on communication materials, letterhead. | 3 |  |
| Management of field Operations |  | 1 |  |
| Consultants | Ex. develop a handbook for Consultants | 1 |  |
