# Supplementary material for: Working toward sustainability: Transitioning HIV programs from a USA-based organization to a local partner in Zimbabwe
Source: PLoS One. 2022 Nov 10;17(11):e0276849. doi: 10.1371/journal.pone.0276849 (PMC9648773; doi:10.1371/journal.pone.0276849)
Supplement: S2 Table — Transition plan t for Zim-TTECH transition. (DOCX) [file pone.0276849.s002.docx]

# S2 Table: Acronyms and Terminology

| **Acronym / Term** | **Definition** |
| --- | --- |
| Award | Financial assistance that provides support or stimulation to accomplish a public purpose. Awards include grants and other agreements in the form of money or property in lieu of money, by the U.S. federal government to an eligible recipient. The term does not include: technical assistance, which provides services instead of money; other assistance in the form of loans, loan guarantees, interest subsidies, or insurance; direct payments of any kind to individuals; and contracts which are required to be entered into and administered under federal procurement laws and regulations (11). |
| C&T | HIV prevention, treatment, care, and support services |
| CORE-Q | Consolidated Criteria for Reporting Qualitative Research |
| HQ | Headquarters |
| HR | Human resources |
| I-TECH | International Training and Education Center for Health |
| LMIC | Low- and middle-income countries |
| NGO | Non-governmental organization |
| PEPFAR | U.S. President’s Emergency Plan for AIDS Relief |
| Prime | “Prime awardee” or “prime recipient”. Refers to a recipient of U.S. federal awards. |
| UZCHS-CTRC | University of Zimbabwe College of Health Sciences Clinical Trials Research Centre |
| VMMC | Voluntary medical male circumcision |
| ZAZIC | A partnership between I-TECH, the Zimbabwe Association of Church Related Hospitals, and Zimbabwe Community Health Intervention Research Project, and UZCHS-CTRC. ZAZIC supports VMMC services in 13 districts across the country (23). |
| ZimPAAC | Zimbabwe Partnership to Accelerate AIDS Control. A consortium that includes AFRICAID, Pangaea Zimbabwe AIDS Trust, UZCHS-CTRC that serves as an implementing and administrative management partner to I-TECH in Zimbabwe. ZimPAAC works in 372 facilities in 17 districts to provide HIV prevention services, testing, active case finding, and linkage to and retention in care and treatment (23). |
| Zim-TTECH | Zimbabwe Technical Assistance, Training and Education Centre for Health |
